# *In Vitro* Growth of *Curcuma longa* L. in Response to Five Mineral Elements and Plant Density in Fed-Batch Culture Systems

**DOI:** 10.1371/journal.pone.0118912

**Published:** 2015-04-01

**Authors:** Rabia F. El-Hawaz, William C. Bridges, Jeffrey W. Adelberg

**Affiliations:** 1 School of Agriculture, Forestry and Environmental Sciences, Clemson University, Clemson, SC, United States of America; 2 Department of Mathematical Sciences, Clemson University, Clemson, SC, United States of America; University of Perugia, ITALY

## Abstract

Plant density was varied with P, Ca, Mg, and KNO_3_ in a multifactor experiment to improve *Curcuma longa* L. micropropagation, biomass and microrhizome development in fed-batch liquid culture. The experiment had two paired D-optimal designs, testing sucrose fed-batch and nutrient sucrose fed-batch techniques. When sucrose became depleted, volume was restored to 5% m/v sucrose in 200 ml of modified liquid MS medium by adding sucrose solutions. Similarly, nutrient sucrose fed-batch was restored to set points with double concentration of treatments’ macronutrient and MS micronutrient solutions, along with sucrose solutions. Changes in the amounts of water and sucrose supplementations were driven by the interaction of P and KNO_3_ concentrations. Increasing P from 1.25 to 6.25 mM increased both multiplication and biomass. The multiplication ratio was greatest in the nutrient sucrose fed-batch technique with the highest level of P, 6 buds/vessel, and the lowest level of Ca and KNO_3_. The highest density (18 buds/vessel) produced the highest fresh biomass at the highest concentrations of KNO_3_ and P with nutrient sucrose fed-batch, and moderate Ca and Mg concentrations. However, maximal rhizome dry biomass required highest P, sucrose fed-batch, and a moderate plant density. Different media formulations and fed-batch techniques were identified to maximize the propagation and storage organ responses. A single experimental design was used to optimize these dual purposes.

## Introduction

Liquid culture has inherent advantages for studying mineral nutrition when compared to semi-solid (agar-type) medium [1], [2]. The lack of gradients within the medium and the greater water availability allows higher ranges of concentrations to be effective, and nutrients can be replenished as needed [3]. In micropropagation, liquid cultures simplify plant material transfer, sterilizing and filling vessels, and allows for more rapid growth, as clear tangible benefits to culture on semi-solid agar culture [4]. Labor costs can be further reduced via automation with large-scale bioreactors [5]. However, plants continuously submerged, in high relative humidity and with unbalanced mineral concentrations in the medium show physiological and morphological disorders referred to as hyprehydricity [6]. Temporary immersion techniques reduce abnormality and increase nutrient uptake, with improved tissue quality apparent during greenhouse acclimatization [4].

Micropropagation is usually conducted as a batch culture, with all of the nutrient medium and plant material combined on the first day of culture, and the tissue removed on the last day. Vessel size and medium volume may limit the growth [3]. Time consuming subculture required to maintain growth adds most of the cost to produce micropropagated plants [7]. Fed-batch techniques allow one or more of the medium’s components to be periodically supplemented during a culture cycle [8] increasing the biomass and chemical production of the cell cultures [9], [10], or organ culture [11], [12].

Liquid systems are especially useful for growth and development of plant storage organs [13]. In one example of shoot culture with turmeric, sucrose fed-batch bioreactors allowed *in vitro* production of rhizomes with their dry mass directly related to the sucrose consumed [14]. Dried rhizomes of turmeric (*Curcuma longa* L.), a perennial herb, have economic value as a spice, dye, and a source of therapeutic and bioactive metabolites [15]. Turmeric rhizomes with strong anti-oxidant properties were developed in large volume (2.5 l) bioreactor cultures when sucrose and water were added to prolong the culture period over 15 to 24 weeks [16], [14].

MS medium [17] has been used for the tissue culture of most crops [1], but may not be an optimal formulation in many of these applications [18]. For example on MS medium the inorganic elements P, Ca, Mg, Mn, and B, were deficient in daylily culture [2], and P and Mg were deficient in turmeric culture [20]. Mineral nutrition is a multifactor problem and modern designs with response surface models were effectively used to screen nutrient salt components of MS for micropropagation [19]. Comprehensive strategies to screen groups of salts, based on mineral use [18], [21], or elemental analysis of the foliage tissue [20] have been used for media optimality studies.

The relation between the number of plants, medium volume and nutrient requirement should be considered in specifying optimal nutrient concentrations during a medium formulation experiment. In turmeric micropropagation, plant density and medium volume altered the amounts of nutrients used with different optimal mineral solutions required for high and low density cultures [20]. Sucrose concentration in media affects dry mass and the amount of NH_4_, NO_3_, Ca, Mg, and K taken up [22]. Multiplication and plant growth responses to plant density varied with medium volume and nutrient availability [23]. This prior work was conducted in batch cultures.

Maximizing *in vitro* shoot multiplication and biomass production may have different optimal concentrations in a fed-batch system, when the entire amount of nutrient does not need to be concentrated at initiation. The objectives of this current work with a turmeric rhizome bioreactor system were (1) to determine the main and interactive effects of the mineral nutrients P, Ca, Mg, and KNO_3_ on growth and development (2) to investigate the interactive effects with the process variables, plant density and fed-batch technique (3) to determine how the amount of sucrose and water required to maintain set points varied with the factors above, and (4) to compare the optimal *in vitro* media for turmeric shoot and rhizome growth. Optimal concentrations and combinations of mineral nutrients were formulated to maximize propagation or dried rhizome biomass.

## Materials and Methods

### Plant material

*Curcuma longa* L. accession L 35–1 was supplied by University of Arizona, Southwest Center for Natural Products Research and Commercialization (UA Herbarium 375,742, ARIZ). Quiescent shoot tips were separated from rhizomes then sterilized and initiated in stage I as previously described by Cousins and Adelberg [16]. Stage II buds were maintained during five years by routine subculture (5-week periods) on 33 ml of modified MS liquid medium on a 100 rpm gyratory shaker, in 180 ml cylindrical glass vessels, with Magenta B cap closures (Magenta Corp, Chicago IL, USA), 1 μM BA, 60 g sucrose per liter, pH 5.7. Vessels were incubated at 23±2ºC under cool white fluorescent lights with the intensity of 30 μmol m^2^s^-1^ photosynthetically active radiation (PAR) for 16 hours per day. Prior to the experiment, buds were assigned to treatment media based on method previously described [20] with plants sub-cultured 3 times to acclimatize to treatment conditions prior to bioreactor culture.

### Treatment media in bioreactors

Treatment media were prepared similar to MS stage II media (above) except with 5% m/v sucrose, 3 μM BA, and 5 mM ammonium sulfate. The combinations of phosphorous (1.25, 3.75, and 6.25 mM), calcium (3, 6, and 9 mM), magnesium (1.5, 3, and 4.5 mM), potassium nitrate (20, 40, and 60 mM), and plant density levels of 6, 12, and 18 buds/vessel were chosen based on D-optimal criteria [24] as shown in (S1 Table). The influence of the minerals as ions (except KNO_3_) on plant responses were tested to avoid the confounding effect of cation and anion pairs in salts [19], and sulfate was used as a counter ion to maintain charge balance in different media solution [20]. Media pH was measured but not controlled.

The trimmed buds were transferred to 35 pairs of bioreactor vessels as specified in S1 Table. Each vessel (2.5 l Liquid Lab Vessels, Southern Sun Inc., Hodges, SC) contained 200 ml of treatment medium. The entire experiment was duplicated for the sucrose fed-batch (SF) and the nutrient sucrose fed-batch (NSF) process. The bioreactors were set on an intermittent immersion rocker system [16] one rotation per minute, for 22-week experimental period.

### Fed-batch techniques

The amount of sucrose and water added during sucrose fed-batch (SF) was calculated by the known addition techniques of Cousins and Adelberg [14]. Sucrose concentration was measured by a refractometer (Atago Model N10, Atago Instruments Ltd., Tokyo, Japan) before (Sb) and after (Sa) the aseptic addition of 10 ml of 20% m/v sucrose solution (S2 Table). Estimates of media volume remaining ml (Vr), volume used ml (Vu), the concentration of sugar used % m/v (Su), and the mass of the sugar used g (Sg) were made with the following equations:

$$Vr\;=\;\left(200-Sa\times 10\right)÷\left(Sa-Sb\right)$$

Vu = 190-Vr

$$Su\;=\;\left\{1000-Sa\times \left(Vr+10\right)\right\}÷Vu$$

$$Sg\;=\;\left(Vu\times Su\right)÷100$$

A sterile sucrose solution was prepared for each vessel to restore 5% m/v sucrose and 200 ml medium volume. With NSF group, in addition to sucrose solution, volumes of double concentration of the factors’ macronutrient and MS micronutrient solutions were required to restore set points (Vn). Spent media samples (Vs) were taken before adding 20% m/v sucrose solution, to NSF vessels. Flow Injection Analyzers (FIAlab-2500; FIAlab instruments, Inc., Bellevue, WA, USA) was used to determine nitrate concentration. Thermo Jarrell Ash Model 61E Inductively Coupled Plasma (ICP; Analytical West, Inc., Corona, CA, USA) was used to measure elements concentrations (P, K, Ca, Mg, Mn, S, Fe, B, Zn, Na, Cl, and Cu) in the spent media (Agricultural Chemical Service of Clemson University). Manganese was chosen as a standard element to estimate minerals used based on the correlation between Mn used, and other minerals (S3 Table). The amount of Mn (Mnu) required restoring set-point of 5.5 ppm Mn was estimated by these equations:

$$Mnu\;=\;\left(5.5\times 200\right)-\left(Mnm\times Vr\right)$$

$$Vn\;=\;Mnu÷\left(5.5\times 2\right)$$

Where Mnm = Mn measured. Water volume ml as (Vw) which required restoring set-point of medium volume (200 ml) was calculated by this equation:

$$Vw\;=\;\left(200+Vs\right)-\left(Vu+Vr\right)$$

### Plant Measurements

Plants were removed from the vessel at the 22^nd^ week. Multiplication ratio (number of harvested plants / initial buds) and fresh and dry biomass of whole plants, roots, leaves, and rhizomes were measured (S4 Table). Tissues were dried in an oven at 80ºC for 72 hours.

### Experimental design and statistical analysis

D-optimal criteria were used to choose 35 *in vitro* treatments combinations (out of 243 possible combinations of the five treatment factors at three levels each). Three true replicates of the treatment combinations were conducted. Sulfate was considered as a covariate with the factors P, Ca, and Mg (S1 Table). The experiment was duplicated for an additional qualitative factor at two levels (fed-batch techniques). Response surface models, including parameters for all the possible linear, quadratic, and interaction terms of the factors, were developed using stepwise forward model building techniques. Parameters were considered significant and included in the final model when the null hypothesis (H_o_: model parameter = 0) test produced a *P*-value <0.01. The utility of the final model was assessed by a combination of the following criteria, (1) the *R* ^*2*^ value, (2) the adjusted *R* ^*2*^ value (*R* ^*2*^_*a*_), (3) the predictive *R* ^*2*^ value (*R* ^*2*^_*P*_), and (4) the *F* statistic and *P*-value. The maximum (or minimum) for water supplementation, sucrose supplementation, multiplication ratio, fresh biomass, and rhizome dry biomass were determined from the final model and used to find the optimal concentrations of the treatment factors (i.e., concentrations that produced the desired means of water supplementation, sucrose supplementation, multiplication ratio, fresh biomass, and rhizome dry biomass). The design, analysis, and graphical visualization were prepared by JMP version 10.0 (SAS Inst., Cary, NC).

## Results and Discussion

Bioreactors may be used for either micropropagation or production of biochemicals in plant biomass. *Curcuma longa* L. multiplication ratio, fresh biomass, and rhizome dry biomass were selected as responses to show the applications for both purposes. Development of storage organs (e.g. micro-rhizomes in turmeric) required maintaining growth over several months by adding water and sucrose on a timely basis [14]. Therefore, optimizing nutrient element formulations for this time frame required sucrose and water supplies to be monitored and controlled.

### Water and sucrose supplementation

**Fig 1:**
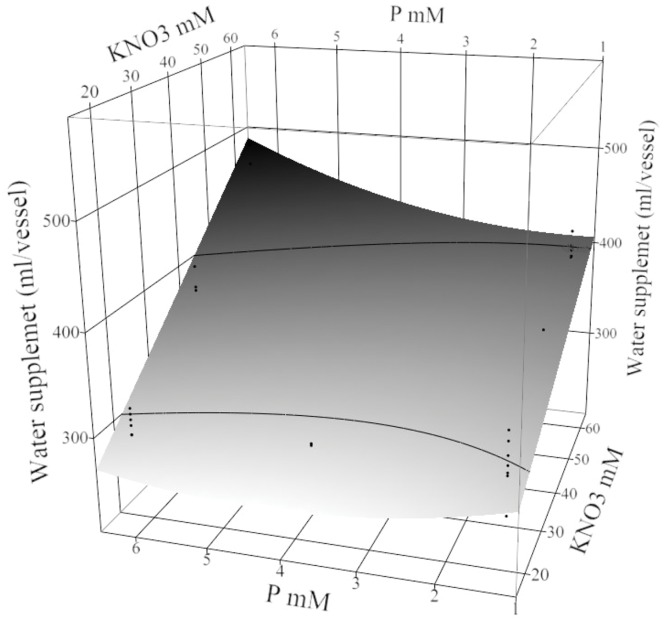
Water supplemented (ml/vessel) to maintain growth over 22 weeks in treatment condition. The response surface plot (with residual data points) shows the effect of P × KNO_3_ on the amount of water (ml/vessel) required to restore medium volume set point of 200 ml per vessel. Other factors were fixed in the sucrose fed-batch technique, with 3 mM Ca, 1.5 mM Mg, and 18 buds/vessel.

The amount of sucrose and water needed in the fed-batch technique was affected by the treatment factors in this experiment. Potassium nitrate, the KNO_3_ × P interaction, the KNO_3_ × Ca interaction, and plant density (S5 Table) all significantly affected water use. Water use increased with increasing KNO_3_ concentration. Water use also increased as P increased at the highest concentration of KNO_3_. For example, at 60 mM KNO_3_ and 6.25 mM P the water supplement was maximized to 471±23 ml (Fig. 1) when other factors remained in the optimal concentrations (18 buds/vessel, 3 mM Ca, 1.5 mM Mg, in SF) and plants required the most water (S5 Table).

**Fig 2:**
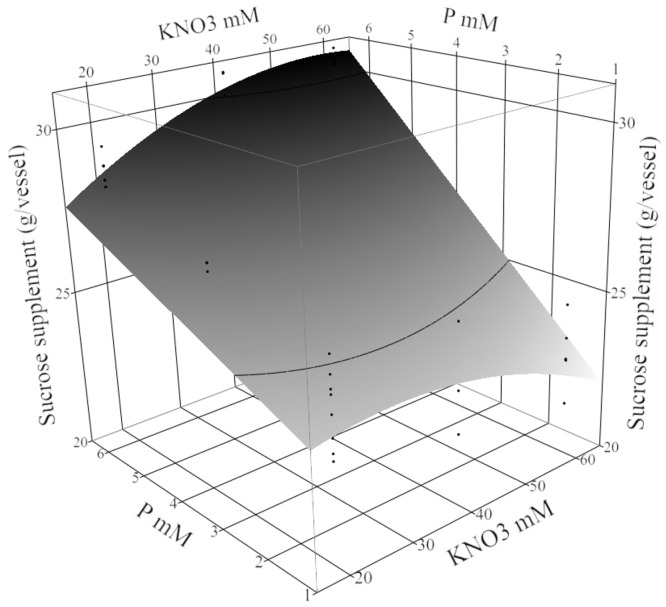
Sucrose supplemented (g/vessel) to maintain growth over 22 weeks in treatment conditions. The response surface plot (with residual data points) shows the effect of P × KNO_3_ on the amount of sucrose (g/vessel) required to restore sucrose set point 5% m/v. Other factors were fixed in the nutrient sucrose fed-batch technique, 3 mM Ca, 3.56 mM Mg, and 18 buds/vessel.

However, the amount of sucrose supplemented was significantly affected by P, plant density, P × KNO_3_ interaction, fed-batch technique, and P × plant density interaction (S6 Table). Phosphorous was the most important factor based on the effect size as measured by the ANOVA mean square. The P × KNO_3_ interaction showed the greatest sucrose addition was needed when KNO_3_ increased at the highest concentrations of P (6.25 mM). However, at 1.25 mM P, a greater sucrose addition was needed when potassium nitrate was decreased. With P and KNO_3_ at the highest concentration and the highest plant density, 30±1 g/vessel of sucrose was needed to maintain set point with NSF (Fig. 2).

It is apparent that increased macronutrients (N, P, and K) and plant density increased turmeric’s need for water and sucrose in the bioreactor system. It is likely that if water and sucrose had not been adjusted in accordance with use, growth responses would have been diminished by sub-optimal or super-optimal amounts of water or sucrose. For example, turmeric growth was reduced at 8% m/v sucrose in liquid MS medium, compared to 4–6%, [25] and a static constant feed sucrose protocol would result in some of low P medium treatments being inhibited by super-optimal sucrose concentrations.

### Multiplication ratio

**Fig 3:**
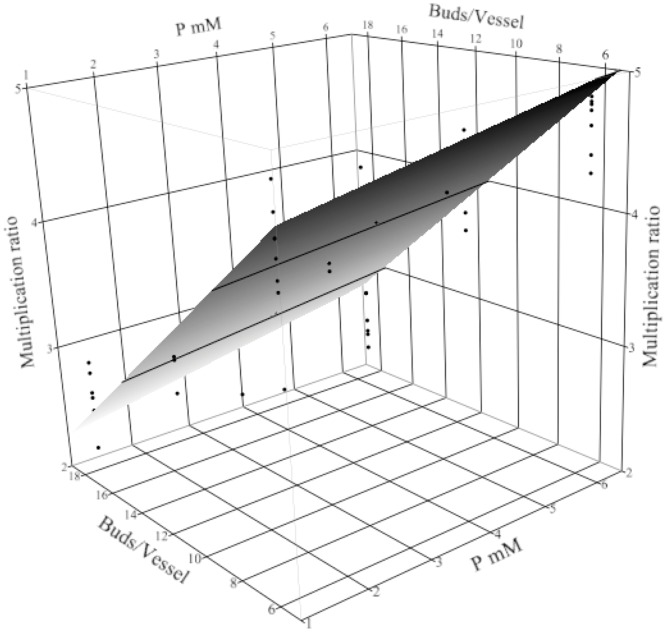
Turmeric multiplication ratio over 22 weeks in treatment conditions. The response surface plot (with residual data points) shows multiplication ratio of *Curcuma longa* L. as effected by the initial plant density (buds/vessel) and P concentration. Other factors were fixed in the nutrient sucrose fed-batch technique, with 3 mM Ca, and 20 mM KNO_3_.

Plant density, P, and the fed-batch technique were the most important factors in the multiplication ratio model (S7 Table). The highest multiplication ratio occurred when the plant density was lowest (6 buds per vessel). In sugarcane [26], hosta [27], garlic [28], and palm culture [29], the multiplication increased with lowered plant density. Increasing P from 1.25 mM, which is the concentration of P in the standard MS medium and the lowest concentration tested to 6.25 mM P produced more plants. Nutrient sucrose fed-batch cultures enhanced plant multiplication and the combined effects of P and plant density had the greatest response when P had the highest concentration (6.25 mM) and plant density was the lowest, 6 buds/vessel (Fig. 3). In a similar study in batch culture of turmeric, the fewest buds had the most the multiplication (4.8-fold) with P increased to 4.11 mM [20].

**Fig 4:**
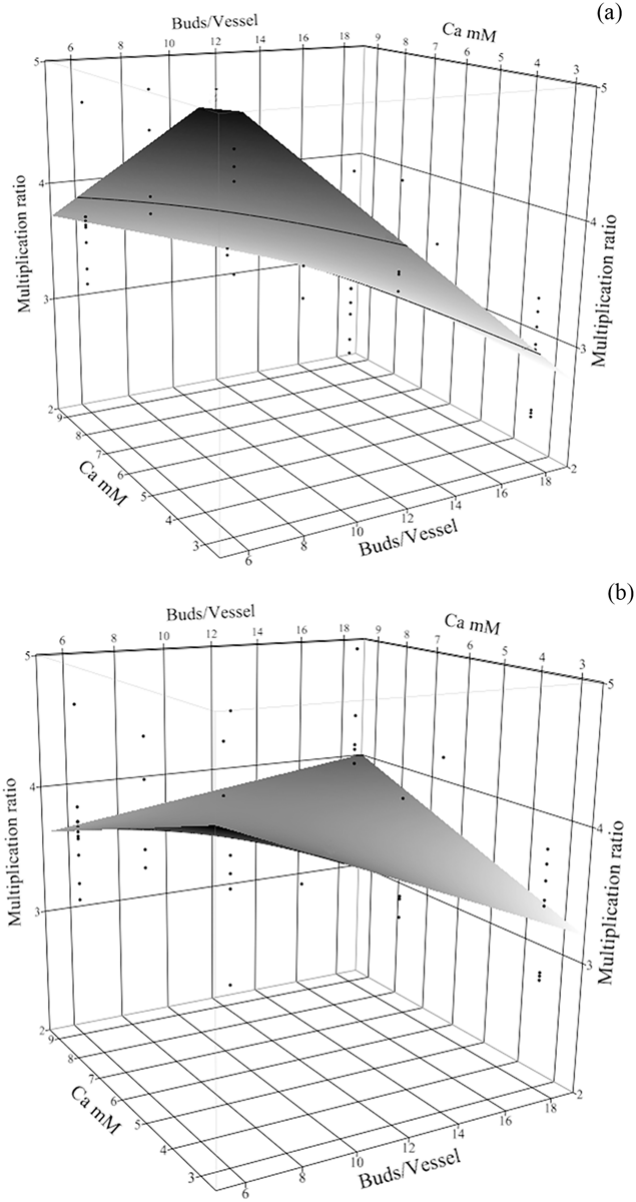
The interaction of plant density and mineral concentrations affected turmeric multiplication ratio. The response surface plots (with residual data points) shows multiplication ratio of *Curcuma longa* L. as effected by the interaction of initial plant density (buds/vessel) × Ca concnentraion when other factors were fixed in the nutrient sucrose fed-batch technique, with 6.25 mM P and (a) 20 mM KNO_3_ and (b) 60 mM KNO_3_.

Similar to previous work [20] in batch culture of turmeric, the optimal concentration of Ca and KNO_3_ for multiplication depended upon the plant density (S7 Table). Few buds and low Ca concentration allowed greatest multiplication (Fig. 4). In 20 mM KNO_3_, raising plant density or Ca caused a great reduction in multiplication (from 5- to 3-fold) as shown in Fig. 4a. But in 60 mM KNO_3_, 18 buds/vessel yielded a moderate multiplication ratio 4-fold. At high KNO_3_ (60 mM) the flattened contour surface indicates more freedom to vary parameters. For example, when the greatest number of plants is preferred, then 60 mM KNO_3_ with high Ca will allow large initial density to yield around 4-fold without causing any reduction in multiplication due to the high density (Fig. 4b). This is similar to the results where the Ca × KNO_3_ interaction allowed the greatest production of new plants in high-density culture when the amount of Ca and KNO_3_ increased in batch culture [20]. Although we could optimize the multiplication for very few plants (6 buds/vessel), it may be more productive to accept moderate multiplication for higher population density (18 buds/vessel). For example, 5-fold multiplication with 6 initial buds yields 30 plants, while 4-fold with 18 initial buds yields 72 plants.

### Fresh biomass

**Fig 5:**
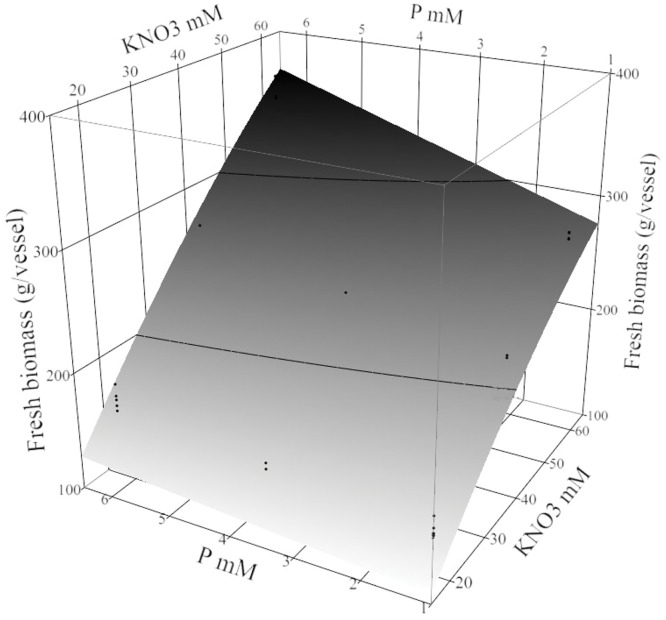
Turmeric fresh biomass (g/vessel) over 22 weeks in treatment conditions. The response surface plot (plus residual data points) shows *Curcuma longa* L. fresh biomass (g/vessel) was affected by P × KNO_3_ interaction. Other factors in the nutrient sucrose fed-batch technique were fixed with 18 buds/vessel, 3 mM Ca, and 3 mM Mg.

Turmeric biomass was affected by fed-batch techniques, and the KNO_3_ × fed-bath interaction, KNO_3_, the P × fed-batch interaction, and the initial plant density (S8 Table). It was important to note that during NSF, other nutrients (S, Fe, Mn, Zn, B, Cl, Na, Mo, and Cu) that were not independent treatment factors appeared to interact with KNO_3_ to produce the greatest biomass. Similarly the response to P was driven by KNO_3_, and since P was modeled as an independent variable, these effects were presented graphically (Fig. 5). At 20 mM KNO_3_, P has a low impact, where the fresh biomass had increased from 131±14 to 156±14 g/vessel (S8 Table). However the increase in KNO_3_ at low P (1.25 mM) raised the fresh biomass to 269±14 g/vessel. Combining the highest concentration of P and KNO_3_ yielded the greatest mass (345±14 g/vessel). In high-density cultures, increasing the biomass required high KNO_3_ (S8 Table). However, decreasing Ca concentration from 9 to 3 mM slightly increased the fresh biomass at 60 mM KNO_3_.

### Rhizome dry biomass

The harvested portion of turmeric is the rhizome and the quality of turmeric is conferred by the concentration of secondary metabolites in rhizome dry biomass [15]. A normal characteristic yellow color, aromatic odor, primary and secondary rhizomes, and stolon structures were observed in these bioreactor grown plants.

Optimized dry biomass of rhizomes in this system had a very different response from multiplication and biomass. The most important factors were P, fed-batch techniques, and plant density (S9 Table). Increased P increased the rhizome dry biomass and SF produced greater dry biomass than NSF (10±1.6 g/vessel vs. 5±1.8 g/vessel).

**Fig 6:**
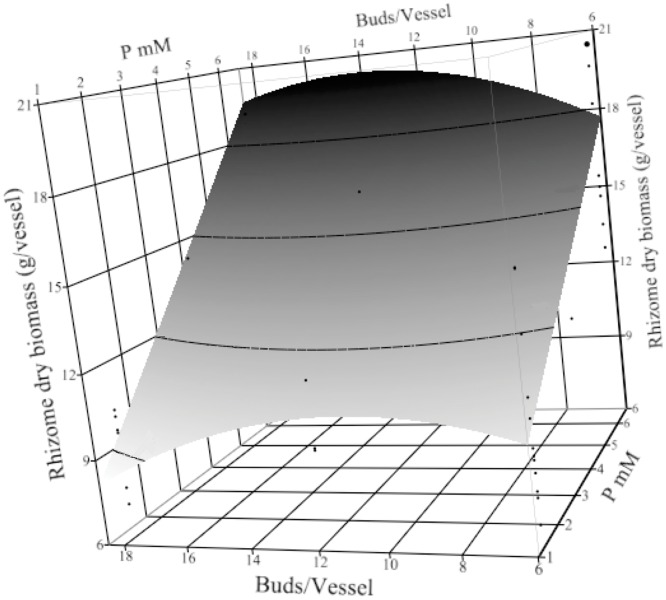
Turmeric rhizome dry biomass (g/vessel) over 22 weeks in treatment conditions. The response surface plot (with residual data points) shows rhizome dry biomass of *Curcuma longa* L. was affected by plant density (buds/vessel) and P concentration. Other factors are fixed in the sucrose fed-batch technique, with 9 mM Ca, 4.5 mM Mg, and 36 mM KNO_3_.

Sucrose fed-batch (with incipient nutrient depletion) may induce plants to start translocation of carbohydrates and other solutes from the leaves, which increased the dry biomass of storage organs [16]. In a similar study turmeric in SF system over 23 weeks, the leaf fresh biomass was reduced after 15 weeks while the rhizome dry biomass increased for the entire 23 weeks [14]. Rhizome biomass increased with plant density up to 14 buds/vessel (Fig. 6), but higher densities did not increase rhizome mass.

Calcium and Mg had a role in rhizome dry biomass and the highest rhizome dry biomass combined the greatest concentration of both Mg and Ca (S9 Table). Unlike fresh biomass, KNO_3_ did not have a major effect on rhizome dry biomass. In prior work with bioreactor grown-turmeric rhizomes, N-deficiency lowered plant biomass, rhizome biomass was not affected and phenolic compounds were increased [30]. In this work, KNO_3_ was the largest source of inorganic nitrogen (20–60 mM), since NH_4_ was fixed at 5 mM.

### Optimal formulae of turmeric cultures

Different optimal media were shown to optimize *in vitro* turmeric growth depending on the chosen response (Table 1). Two formulae were developed for high and low plant densities (6 and 18 buds/vessel) to maximize plant multiplication ratio by modifying the concentrations of KNO_3_ and Ca. Low density required lowest concentrations of Ca and KNO_3_ and high density required the highest concentrations. Other work with batch culture of turmeric shows a low plant density formula maximized turmeric multiplication in low Ca and KNO_3_ concentrations, and in the high plant density culture the production of new plants increased with the increased concentrations of Ca and KNO_3_ [20].

**Table 1:** Maximum growth responses for multiplication, fresh biomass and rhizome dry mass were observed in different fed-batch techniques, mineral concentrations and plant densities.

Response	Maximum Value	Fed-batch techniques	Buds/ Vessel	P	Ca	Mg	KNO_3_
**Multiplication ratio (low density)**	5×	NSF	6	6.25	3	-	20
**Multiplication ratio (high density)**	4×	NSF	18	6.25	9	-	60
**Plant fresh biomass**	345 g	NSF	18	6.25	3	3	60
**Rhizome dry biomass**	19.5 g	SF	14	6.25	9	4.5	36

Minerals concentration in milimolar. NSF stands for Nutrients Sucrose Fed-batch; SF stands for Sucrose Fed-batch.

The optimal formula for fresh biomass for high plant density used the highest concentrations of P and a moderate concentration of KNO_3_ (6.25 and 60 mM) respectively (Table 1). Applying the maximal biomass model in S7 Table, would yield a multiplication ratio to 3.4±0.4 fold in the high-density and is not optimal for multiplication.

Rhizome dry biomass has a different set of optimal conditions and was antagonized by NSF technique that was better suited for propagation. The rhizome dry biomass was estimated to be 3±1.8 g/vessel from multiplication formula (6 buds/vessel, 6.25 mM P, 3 mM Ca, and 20 mM KNO_3_ in NSF) and to 5±1.8 g/vessel from fresh biomass formula (18 buds/vessel, 6.25 mM P, 3 mM Ca, 3 mM Mg, and 60 mM KNO_3_ in NSF). However, maximum rhizome dry biomass (19.5±1.8 g/vessel) was produced from 14 buds/vessel, 6.25 mM P, 9 mM Ca, 4.5 mM Mg, and 36 mM KNO_3_ in SF (Table 1). The same formula reduced the rhizome dry mass to about 13±1.8 g/vessel in NSF. Sucrose fed-batch (SF) is a simper technique than NSF and also yields higher dry rhizome biomass than NSF.

Five months of bioreactor culture required 26.8±1 g/vessel of sucrose to produce 19.5±1.8 g of rhizome dry mass in SF with 6.25 mM P (and other factors maximized by rhizome dry biomass formula Table 1). Phosphorous played an important role to increase sucrose conversion to dry biomass. With 1.25 mM P (MS level), only 10.5±1.8 g/vessel of rhizome dry tissue was produced from 22.4±1 g/vessel sucrose in SF, and 8.3±1.8 g/vessel of rhizome dry tissue required 23.9±1 g/vessel sucrose in NSF (S6 and S9 Table). Bioconversion of sucrose to turmeric rhizome creates value and the efficiency of that conversion was greatly influenced by the concentrations of the mineral nutrients.

## Conclusion

Plant growth and development in large vessels (2.5 l) over a long period in treatment conditions (5-months) allowed significant expression of the effects of the treatment factors on turmeric growth. The models presented here have better predictive value (R^2^_p_) than the models prior work in small vessels (180 ml) batch cultures over 35-days with similar treatment factor combinations [20]. The sparsely sampled design space was allowed accurate modeling of complex relationships in fed-batch bioreactors. Prior work with SF bioreactors shows sucrose conversion to rhizome mass in turmeric can be scaled from batch to fed-batch over longer periods of time [14]. Similarly, NSF technique, 2.5 l vessels, and experimental designs used in this work could predict mineral use and optimal nutrition for larger-scale bioreactors.

Potassium nitrate and P induce fresh and dry plant biomass when water and sucrose are supplied in adequate amounts through a fed batch process. NSF technique is recommend when 1) propagating at low plant density which is appropriate for increasing the number of elite plants *in vitro*, and 2) using high plant density required more nutrients to produce more biomass. However, rhizomes have a different optimal medium than multiplication ratio and fresh biomass production with the incipient nutrients stress of SF necessary to maximize rhizome dry biomass. Optimal concentrations of the mineral nutrients in a media formulation changes with the desired response and the process used.

## Supporting Information

S1 Table Treatment combinations of P, Ca, Mg, KNO_3_, and plant density (buds/vessel) of turmeric included three true replicates. Sulfate was a covariate and the entire design was repeated for both nutrient sucrose fed-batch and sucrose fed-batch techniques.
(DOCX)

S2 Table Fed-batch techniques. (XLSX)

S3 Table The correlation matrix of the minerals used in the first cycle. Use of 13 elements by turmeric tissue during 8-weeks in treatment conditions was correlated with the relationships among pairs of elements indicated by a matrix of coefficients.
(TIFF)

S4 Table Plant measurements. (XLSX)

S5 Table The model of water supplemented (ml/vessel) to maintain growth over 22 weeks in treatment condition. The final model had *R* ^*2*^ = 0.768, *R* ^*2*^_*a*_ = 0.686, and *R* ^*2*^_*p*_ = 0.555, and *F* statistic = 9.419 (*P*-value <0.0001. NSF stands for Nutrients Sucrose Fed-batch.
(DOCX)

S6 Table The model of sucrose supplemented (g/vessel) to maintain growth over 22 weeks in treatment conditions. The final model had *R* ^*2*^ = 0.922, *R* ^*2*^_*a*_ = 0.891, and *R* ^*2*^_*p*_ = 0.843, and *F* statistic = 30.235 (*P*-value <0.0001). NSF stands for Nutrients Sucrose Fed-batch.
(DOCX)

S7 Table The model of turmeric multiplication ratio over 22 weeks in treatment conditions. The final model had *R* ^*2*^ = 0.708, *R* ^*2*^_*a*_ = 0.626, and *R* ^*2*^_*p*_ = 0.528, and *F* statistic = 8.611 (*P*-value <0.0001). NSF stands for Nutrients Sucrose Fed-batch.
(DOCX)

S8 Table The model of turmeric fresh biomass (g/vessel) over 22 weeks in treatment conditions. The final model had *R* ^*2*^ = 0.949, *R* ^*2*^_*a*_ = 0.927, and *R* ^*2*^_*p*_ = 0.825, and *F* statistic = 43.204 (*P*-value <0.0001). NSF stands for Nutrients Sucrose Fed-batch.
(DOCX)

S9 Table The model of turmeric rhizome dry biomass (g/vessel) over 22 weeks in treatment conditions. The final model had *R* ^*2*^ = 0.758, *R* ^*2*^_*a*_ = 0.664, and *R* ^*2*^_*p*_ = 0.456, and *F* statistic = 8.073 (*P*-value <0.0001). NSF stands for Nutrients Sucrose Fed-batch.
(DOCX)
