# Supplementary material for: In Vitro Growth of Curcuma longa L. in Response to Five Mineral Elements and Plant Density in Fed-Batch Culture Systems
Source: PLoS One. 2015 Apr 1;10(4):e0118912. doi: 10.1371/journal.pone.0118912 (PMC4382179; doi:10.1371/journal.pone.0118912)
Supplement: S1 Table — Sulfate was a covariate and the entire design was repeated for both nutrient sucrose fed-batch and sucrose fed-batch techniques. (DOCX) [file pone.0118912.s001.docx]

| **Buds/Vessel** | **P mM** | **Ca mM** | **Mg mM** | **KNO_3_ mM** | **SO_4_ mM** |
| --- | --- | --- | --- | --- | --- |
| 6 | 1.25 | 3 | 1.5 | 20 | 6.50 |
| 6 | 1.25 | 3 | 3 | 40 | 8.00 |
| 6 | 1.25 | 6 | 4.5 | 60 | 12.50 |
| 6 | 1.25 | 9 | 1.5 | 60 | 12.50 |
| 6 | 1.25 | 9 | 3 | 40 | 15.50 |
| 6 | 1.25 | 9 | 4.5 | 20 | 15.50 |
| 6 | 3.75 | 3 | 4.5 | 60 | 8.25 |
| 6 | 3.75 | 9 | 1.5 | 40 | 11.25 |
| 6 | 6.25 | 3 | 1.5 | 60 | 4.13 |
| 6 | 6.25 | 3 | 4.5 | 20 | 7.13 |
| 6 | 6.25 | 6 | 3 | 20 | 8.50 |
| 6 | 6.25 | 9 | 1.5 | 20 | 10.00 |
| 6 | 6.25 | 9 | 4.5 | 40 | 13.00 |
| 6 | 6.25 | 9 | 4.5 | 60 | 13.00 |
| 12 | 1.25 | 3 | 4.5 | 60 | 9.50 |
| 12 | 1.25 | 6 | 1.5 | 20 | 9.50 |
| 12 | 3.75 | 9 | 4.5 | 20 | 14.25 |
| 12 | 6.25 | 3 | 1.5 | 40 | 4.13 |
| 12 | 6.25 | 3 | 4.5 | 20 | 7.13 |
| 12 | 6.25 | 9 | 3 | 60 | 11.50 |
| 18 | 1.25 | 3 | 1.5 | 60 | 6.50 |
| 18 | 1.25 | 3 | 4.5 | 20 | 9.50 |
| 18 | 1.25 | 9 | 1.5 | 20 | 12.50 |
| 18 | 1.25 | 9 | 3 | 40 | 14.00 |
| 18 | 1.25 | 9 | 4.5 | 60 | 15.50 |
| 18 | 1.25 | 9 | 4.5 | 60 | 15.50 |
| 18 | 3.75 | 3 | 3 | 20 | 6.75 |
| 18 | 3.75 | 6 | 1.5 | 60 | 8.25 |
| 18 | 6.25 | 3 | 1.5 | 20 | 4.13 |
| 18 | 6.25 | 3 | 4.5 | 60 | 7.13 |
| 18 | 6.25 | 3 | 4.5 | 60 | 7.13 |
| 18 | 6.25 | 6 | 4.5 | 40 | 10.00 |
| 18 | 6.25 | 9 | 1.5 | 20 | 10.00 |
| 18 | 6.25 | 9 | 1.5 | 60 | 10.00 |
| 18 | 6.25 | 9 | 4.5 | 20 | 13.00 |
