# Supplementary figures and images for: In Vitro Growth of Curcuma longa L. in Response to Five Mineral Elements and Plant Density in Fed-Batch Culture Systems
Source: PLoS One. 2015 Apr 1;10(4):e0118912. doi: 10.1371/journal.pone.0118912 (PMC4382179; doi:10.1371/journal.pone.0118912)

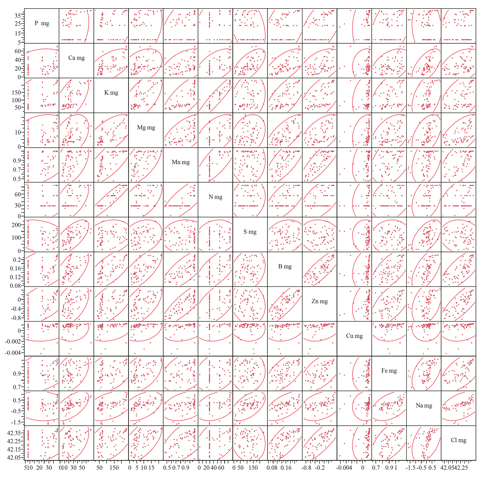

Supplement: S3 Table — Use of 13 elements by turmeric tissue during 8-weeks in treatment conditions was correlated with the relationships among pairs of elements indicated by a matrix of coefficients. (TIFF) [file pone.0118912.s003.tiff]
