# Supplementary material for: In Vitro Growth of Curcuma longa L. in Response to Five Mineral Elements and Plant Density in Fed-Batch Culture Systems
Source: PLoS One. 2015 Apr 1;10(4):e0118912. doi: 10.1371/journal.pone.0118912 (PMC4382179; doi:10.1371/journal.pone.0118912)
Supplement: S5 Table — The final model had R 2 = 0.768, R 2 a = 0.686, and R 2 p = 0.555, and F statistic = 9.419 (P-value <0.0001. NSF stands for Nutrients Sucrose Fed-batch. (DOCX) [file pone.0118912.s005.docx]

| **Model terms** | **Parameter estimate** | ***P*-value of t-test** | **Mean square** |
| --- | --- | --- | --- |
| KNO_3_ mM | 1.4122±0.2196 | <0.0001 | 22812.195 |
| P × KNO_3_ mM | 0.3561±0.0804 | <0.0001 | 10811.856 |
| Ca × KNO_3_ mM | -0.2650±0.0779 | 0.0016 | 6386.364 |
| Buds/Vessel | 2.1002±0.6246 | 0.0018 | 6236.240 |
| NSF × KNO_3_ mM | 0.5220±0.2041 | 0.0148 | 3607.652 |
| P mM | 3.5208±1.4171 | 0.0176 | 3405.090 |
| NSF × Buds/Vessel | -1.3947±0.6130 | 0.0288 | 2855.128 |
| Mg × KNO_3_ mM | -0.3163±0.1614 | 0.0576 | 2118.326 |
| NSF | -5.6030±3.3997 | 0.1078 | 1498.296 |
| (P mM)^2^ | 2.9495±1.8434 | 0.1181 | 1412.218 |
| Ca mM | -1.8598±1.2451 | 0.1437 | 1230.768 |
| NSF × Mg mM | 3.2262±2.5977 | 0.2221 | 850.905 |
| Mg mM | 2.3164±2.6296 | 0.3841 | 428.048 |
