# Supplementary material for: In Vitro Growth of Curcuma longa L. in Response to Five Mineral Elements and Plant Density in Fed-Batch Culture Systems
Source: PLoS One. 2015 Apr 1;10(4):e0118912. doi: 10.1371/journal.pone.0118912 (PMC4382179; doi:10.1371/journal.pone.0118912)
Supplement: S6 Table — The final model had R 2 = 0.922, R 2 a = 0.891, and R 2 p = 0.843, and F statistic = 30.235 (P-value <0.0001). NSF stands for Nutrients Sucrose Fed-batch. (DOCX) [file pone.0118912.s006.docx]

| **Model terms** | **Parameter estimate** | ***P*-value of t-test** | **Mean square** |
| --- | --- | --- | --- |
| P mM | 0.7699±0.0589 | <0.0001 | 163.1830 |
| Buds/Vessel | 0.2815±0.0260 | <0.0001 | 111.7560 |
| P × KNO_3_ mM | 0.0177±0.0033 | <0.0001 | 26.1961 |
| NSF | 0.6202±0.1429 | 0.0001 | 18.0093 |
| Buds/Vessel × P mM | 0.0355±0.0110 | 0.0028 | 9.8469 |
| (Buds/Vessel)^2^ | -0.0277±0.0102 | 0.0104 | 6.9959 |
| (Mg mM)^2^ | -0.3302±0.1788 | 0.0730 | 3.2623 |
| NSF × Mg mM | 0.1659±0.1041 | 0.1199 | 2.4278 |
| Ca mM | -0.0782±0.0527 | 0.1466 | 2.1059 |
| (KNO_3_ mM)^2^ | -0.0015±0.0010 | 0.1519 | 2.0498 |
| NSF × Buds/Vessel | -0.0342±0.0255 | 0.1885 | 1.7188 |
| KNO_3_ mM | 0.0197±0.0151 | 0.2006 | 1.6262 |
| Buds/Vessel × KNO_3_ mM | 0.0017±0.0015 | 0.2470 | 1.3247 |
| Mg mM | 0.0434±0.1317 | 0.7434 | 0.1041 |
