# Supplementary material for: In Vitro Growth of Curcuma longa L. in Response to Five Mineral Elements and Plant Density in Fed-Batch Culture Systems
Source: PLoS One. 2015 Apr 1;10(4):e0118912. doi: 10.1371/journal.pone.0118912 (PMC4382179; doi:10.1371/journal.pone.0118912)
Supplement: S7 Table — The final model had R 2 = 0.708, R 2 a = 0.626, and R 2 p = 0.528, and F statistic = 8.611 (P-value <0.0001). NSF stands for Nutrients Sucrose Fed-batch. (DOCX) [file pone.0118912.s007.docx]

| **Model terms** | **Parameter estimate** | ***P*-value of t-test** | **Mean square** |
| --- | --- | --- | --- |
| Buds/Vessel | -0.0658±0.0128 | <0.0001 | 6.2754 |
| P mM | 0.1284±0.0295 | <0.0001 | 4.4883 |
| NSF | 0.3058±0.0706 | 0.0001 | 4.4524 |
| Buds/Vessel × Ca mM | 0.0153±0.0050 | 0.0043 | 2.1803 |
| Buds/Vessel × KNO_3_ mM | 0.0022±0.0007 | 0.0058 | 2.0219 |
| Ca mM | -0.0648±0.0255 | 0.0154 | 1.5256 |
| KNO_3_ mM | -0.0127±0.0071 | 0.0823 | 0.7556 |
| (KNO_3_ mM)^2^ | 0.0008±0.0004 | 0.0935 | 0.7024 |
| P × KNO_3_ mM | 0.0029±0.0017 | 0.1114 | 0.6419 |
| Ca × KNO_3_ mM | 0.0025±0.0015 | 0.1083 | 0.6305 |
| NSF × Buds/Vessel | -0.0153±0.0127 | 0.2355 | 0.3449 |
