# Supplementary material for: In Vitro Growth of Curcuma longa L. in Response to Five Mineral Elements and Plant Density in Fed-Batch Culture Systems
Source: PLoS One. 2015 Apr 1;10(4):e0118912. doi: 10.1371/journal.pone.0118912 (PMC4382179; doi:10.1371/journal.pone.0118912)
Supplement: S8 Table — The final model had R 2 = 0.949, R 2 a = 0.927, and R 2 p = 0.825, and F statistic = 43.204 (P-value <0.0001). NSF stands for Nutrients Sucrose Fed-batch. (DOCX) [file pone.0118912.s008.docx]

| **Model terms** | **Parameter estimate** | ***P*-value of t-test** | **Mean square** |
| --- | --- | --- | --- |
| NSF | 23.2889±1.9842 | <0.0001 | 25443.597 |
| KNO_3_ mM | 2.4019±0.2137 | <0.0001 | 23314.684 |
| NSF × KNO_3_ mM | 1.1519±0.1162 | <0.0001 | 18131.503 |
| P mM | 7.5218±0.8319 | <0.0001 | 15098.882 |
| Buds/Vessel | 2.6858±0.3555 | <0.0001 | 10542.856 |
| P mM × KNO_3_ mM | 0.2564±0.0476 | <0.0001 | 5346.780 |
| Buds/Vessel × KNO_3_ mM | 0.0741±0.0215 | 0.0015 | 2178.297 |
| Ca mM × KNO_3_ mM | -0.1115±0.0484 | 0.0273 | 979.530 |
| NSF × Ca mM | -1.6469±0.7250 | 0.0294 | 953.124 |
| Buds/Vessel × P mM | 0.3309±0.1638 | 0.0511 | 753.602 |
| (Mg mM)^2^ | -4.6920±2.4885 | 0.0677 | 656.589 |
| Ca mM | -1.3133±0.7457 | 0.0870 | 572.848 |
| (KNO_3_ mM)^2^ | -0.0238±0.0149 | 0.1205 | 467.905 |
| NSF × P mM | -1.1759±0.8509 | 0.1757 | 352.754 |
| Mg mM | -2.0834±1.8582 | 0.2698 | 232.754 |
