# Supplementary material for: In Vitro Growth of Curcuma longa L. in Response to Five Mineral Elements and Plant Density in Fed-Batch Culture Systems
Source: PLoS One. 2015 Apr 1;10(4):e0118912. doi: 10.1371/journal.pone.0118912 (PMC4382179; doi:10.1371/journal.pone.0118912)
Supplement: S9 Table — The final model had R 2 = 0.758, R 2 a = 0.664, and R 2 p = 0.456, and F statistic = 8.073 (P-value <0.0001). NSF stands for Nutrients Sucrose Fed-batch. (DOCX) [file pone.0118912.s009.docx]

| **Model terms** | **Parameter estimate** | ***P*-value of t-test** | **Mean square** |
| --- | --- | --- | --- |
| P mM | 0.9855±0.1871 | <0.0001 | 257.4770 |
| NSF | -2.1224±0.4461 | <0.0001 | 210.1574 |
| Buds/Vessel | 0.2296±0.0817 | 0.0080 | 73.2802 |
| Ca mM × Mg mM | 0.3049±0.1254 | 0.0201 | 54.9128 |
| Mg mM | 0.9439±0.4098 | 0.0272 | 49.2646 |
| Buds/Vessel × Ca mM | -0.0700±0.0304 | 0.0273 | 49.1909 |
| (KNO_3_ mM)^2^ | -0.0076±0.0033 | 0.0293 | 47.8207 |
| NSF × P mM | -0.4098±0.1853 | 0.0334 | 45.4159 |
| (Mg mM)^2^ | 1.0944±0.5614 | 0.0591 | 35.2889 |
| P mM × Mg mM | 0.2358±0.1384 | 0.0970 | 26.9640 |
| Buds/Vessel × P mM | 0.0460±0.0356 | 0.2043 | 15.5231 |
| (Buds/Vessel)^2^ | -0.0394±0.0321 | 0.2270 | 14.0334 |
| Ca mM | 0.1435±0.1675 | 0.3972 | 6.8173 |
| KNO_3_ mM | 0.0388±0.0479 | 0.4227 | 6.1089 |
